# Bidirectional effects of neutrophils on *Streptococcus oralis* biofilms *in vitro*

**DOI:** 10.1080/20002297.2025.2453986

**Published:** 2025-01-23

**Authors:** Basmah M. Almaarik, Rizwan Ali, Paul R. Cooper, Michael R. Milward, Josefine Hirschfeld

**Affiliations:** a Clinical Laboratory Science Department (CLS), College of Applied Medical Sciences (CAMS), King Saud University (KSU), Riyadh, Saudi Arabia; b Periodontal Research Group, Department of Dentistry, School of Health Sciences, College of Medicine and Health, University of Birmingham, Edgbaston, UK; c Medical Research Core Facility and Platforms, King Abdullah International Medical Research Center (KAIMRC), King Saud Bin Abdulaziz University for Health Sciences (KSAU-HS), Riyadh, Saudi Arabia; d Department of Oral Sciences, Sir John Walsh Research Institute, Faculty of Dentistry, University of Otago, Dunedin, New Zealand

**Keywords:** *Streptococcus oralis*, biofilm, neutrophils, eDNA, periodontitis

## Abstract

**Background:**

*Streptococcus oralis* is a commensal bacterium and an early biofilm coloniser found in the human oral cavity. One of the biofilm matrix constituents is bacterial extracellular DNA (eDNA). Neutrophils are innate immune cells that respond to biofilms, employing antimicrobial mechanisms such as neutrophil extracellular trap (NET) and reactive oxygen species (ROS) release. Here, bidirectional effects of neutrophils on *S.* *oralis* biofilms were investigated.

**Materials and methods:**

Isolated neutrophils were introduced to *S. oralis* biofilms at different stages of biofilm development. Biofilm quantity was assessed by crystal violet technique, confocal microscopy and CFU enumeration. Surface adhesion during shear stress was quantified by spectrophotometry. Bacterial and neutrophil extracellular DNA within biofilms and ROS production were analysed using fluorescence and luminescence assays, and neutrophil-eDNA interactions were investigated by flow cytometry and fluorescence microscopy.

**Results:**

Neutrophils decreased *S. oralis* biofilm quantity transiently and reduced eDNA but did not affect biofilm surface adhesion. Unexpectedly, CFUs were increased by neutrophils. Bacterial DNA was found to co-localise with neutrophil membranes. Neutrophils produced elevated total and intracellular ROS, however, no NETs in response to biofilms.

**Conclusion:**

Neutrophils *in* *vitro* are not excessively activated by *S. oralis* biofilms but are able to reduce biofilm quantity in the short-term, possibly through interfering with eDNA.

## Introduction

Biofilms are three-dimensional complex multicellular communities embedded in an extracellular polymeric substance (EPS). One of the key structures of EPS is extracellular DNA (eDNA), which provides structural stability to the matrix and plays a role in the protection against host immune responses [1,2]. eDNA can be generated via cell lysis, secretion, or cell death, and it undergoes physical and chemical changes from its genomic DNA precursor within the biofilm matrix [3]. eDNA itself has a complex three-dimensional structure that is entangled with other biofilm components, generating a heterogeneous network [4]. Oral and dental biofilms, also termed plaque, are one example of multispecies *in vivo* biofilms. Periodontal diseases, comprised mainly of gingivitis and periodontitis, are considered among the most prevalent infectious-inflammatory diseases worldwide [5].

Gingivitis is characterised by a reversible inflammation of the gingival tissues evoked by supragingival biofilms mainly comprised of Gram-positive and aerobic bacteria [6]. In contrast, the hallmark of periodontitis is an inflammatory host response towards a dysbiotic, mostly Gram-negative anaerobic biofilm residing in periodontal pockets formed through the breakdown of periodontal tissues. This disease, if left untreated, often leads to tooth loss and systemic inflammation [7,8]. Host responses against dental plaque are essential determinants of periodontal disease onset and severity [9]. *Streptococcus spp*. and *Actinomyces spp*. make up a high proportion in oral health-related multispecies biofilms, with *Streptococcus oralis* (*S. oralis*) occurring in the early biofilm development stage [10,11].

The main protective cell for innate host defence in periodontitis is the neutrophil, which is present in large numbers within the gingival crevice and periodontal tissues. Neutrophil influx towards dental plaque and the ability of oral bacteria to trigger phagocytosis, degranulation, and intra-biofilm release of neutrophil extracellular traps (NETs) have been studied extensively [12–14]. In addition, NETs have also recently been identified within dental calculus, where they were demonstrated to induce its formation [15]. Very interestingly, this study also showed that *Streptococcus mutans* biofilms can convert the NET-DNA configuration into one that is resistant to nuclease degradation. Notably, NET release is closely related to reactive oxygen species (ROS) production and ROS were identified as an essential component of most reaction cascades involving the release of NETs [16,17].

Since NETs are composed mainly of DNA fibres, it appears plausible that neutrophil DNA may inadvertently enhance biofilm EPS by contributing to the bacteria-derived eDNA. Few studies have investigated this theory. Mikolai and colleagues analysed NET responses to *S. oralis*, which the authors referred to as a ‘balance-like’ neutrophil response, where neutrophils elicited defence responses against this microorganism without leading to a dysbalance between host defence and tissue destruction, unlike their response to other periodontal species [18].

Although neutrophils play a crucial protective role against periodontal infection and promote wound healing, they are often described as ‘double-edged swords’ due to the significant tissue damage they elicit during the antimicrobial defence [19]. Neutrophils are understood to contribute to periodontal tissue loss during periodontitis. In periodontal health, they contribute to a balanced host response against early colonisers of oral biofilms. However, little is known regarding the effects of neutrophils on biofilm formation and maturation. Understanding this host-biofilm interaction could help develop novel anti-biofilm treatment strategies.

Consequently, this research had two aims: firstly, to investigate whether neutrophils have the ability to positively or negatively impact initial biofilm formation *in vitro* along with possible mechanisms. Secondly, to analyse whether and how these biofilms activate neutrophil responses. Towards this goal, we employed *S. oralis* single-species biofilms, representing early colonisers of dental biofilms. This microorganism was selected as it is found in early dental biofilms as an initial coloniser [10,11,20,21], and due its pivotal role in establishing and maintaining biofilms, the main risk factor for plaque-induced oral diseases.

## Materials and methods

### Isolation of neutrophils

For neutrophil isolation, either fresh peripheral heparinised blood from healthy volunteers at the University of Birmingham, UK (ethics reference: BCHCDent 024.2024), or buffy coats from healthy, unmedicated adult blood bank donors at King Khalid University Hospital (KKUH; E-20-4671) were used. All KKUH blood donors’ selection criteria are based on the Gulf Cooperation Council (GCC) guidelines for the production and quality control of blood products. Donated blood was separated using the Revos® automated blood processing system (TerumoBCT, Japan), and buffy coats were used for neutrophil isolation.

Neutrophils were isolated from fresh blood or from buffy coats at room temperature under sterile conditions using a class II biological safety cabinet. Isolation was performed using Percoll gradients as described previously [22,23]. All reagents used were stored at 4°C and pre-warmed to room temperature before use. ROS and NET quantification assays were carried out at the University of Birmingham using fresh blood, whilst all other experiments were completed at King Saud University, SA, using blood bank donors’ neutrophils.

### Neutrophil survival and biofilm growth in different bacterial growth media

To determine the effect of neutrophils on biofilm development, we aimed to identify a growth medium that would support both biofilm and neutrophil survival. After neutrophil isolation and counting, 1 × 10^6^ cells were suspended in 1 ml of each media type and incubated for 4 h at 37°C in 5% CO_2_. Media tested were 1) RPMI 1640 medium (positive control; Gibco, Grand Island, NY, USA) + 10% fetal bovine serum (FBS; 10099141 Gibco; Thermo Scientific, Baskingstoke, UK), 2) RPMI 1640, 3) tryptic soy broth (TSB), 4) Schaedler anaerobe broth (SB: Oxoid, Basingstoke, UK), and ultrapure deionised water was used as a cell death-inducing control. All samples were counted under a microscope using a haemocytometer after staining with trypan blue.

### Bacterial culture and biofilm formation

*Streptococcus oralis* (ATCC 9811) was provided by the by the Periodontal Research Group at Birmingham Dental School and was originally purchased from the American Type Culture Collection (ATCC). *S. oralis* was grown on tryptic soy agar (TSA; HiMedia Laboratories Pvt. Ltd, Mumbai, India) and incubated at 37°C in a 5% CO_2_ atmosphere overnight. Purity of the culture was regularly monitored by Gram staining. For biofilm development, one bacterial colony from an overnight culture was transferred and inoculated into TSB and incubated overnight at 37°C on a rotary shaker to reach the plateau phase according to a previously obtained growth curve (**Suppl. Fig. S1)**. A dilution of fresh overnight culture was prepared (OD_600_ of 0.15 for all experiments).

Biofilm formation was undertaken in untreated 24-well plates (Nunclon, Wiesbaden, Germany). Circular 12 mm coverslips were coated for 10 min with poly-L-lysine (P4832, Invitrogen, Renfrew, UK) at room temperature, allowed to dry and transferred to culture wells [24]. Subsequently, 1 ml aliquots of the diluted bacterial suspension in TSB were added to wells in triplicates, and wells containing 1 ml sterile growth medium were included as blank controls. Culture plates were incubated at 37°C in 5% CO_2_ for 48 h. Media was exchanged daily by removing spent media carefully without disturbing the biofilm, and 1 ml of fresh sterile pre-warmed broth was slowly added to the side of the well to prevent biofilm disruption.

The effect of neutrophils on *S. oralis* biofilms was investigated by adding neutrophils at different time points during the 48 h culture period at: 1) before biofilm formation, 2) after 24 h and 3) after 46 h of biofilm formation for 2 h. For condition 1) before biofilm formation, 1 ml of 1 × 10^6^ neutrophils suspended in TSB were added to the wells and allowed to settle for 30 min at room temperature, followed by careful removal of TSB. It was verified that all cells had settled in the culture and sufficiently adhered by performing a cell count in the removed media, subsequently 1 ml of *S. oralis* overnight suspension was added to each well. Control biofilms without neutrophils in triplicate were run in all biofilm experiments.

For condition 2) after 24 h, neutrophils were supplemented during media change after 24 h. For condition 3), neutrophils were added after 46 h and incubated with the biofilms for a further 2 h. All *S.oralis* biofilms in this study were cultured for a total of 48 h with daily media changes as described above. As controls, neutrophils were fixed overnight in 4% paraformaldehyde (*p*-6148 Merck, Darmstadt, Germany) and then washed 3 times with PBS before adding these to the wells, in order to determine the effect of non-viable neutrophils on bacterial biofilms.

## Imaging of neutrophils and biofilms

To assess the ability of neutrophils to produce NETs in response to *S. oralis*, confocal laser scanning and fluorescence microscopy (CLSM: Zeiss Imager Z2 microscope with LSM 780 CLSM and Zeiss Zen 2012 Software [Zeiss, Jena, Germany]) and a Zeiss Axiovert inverted 200 M fluorescence microscope were used for imaging. Scanning electron microscope (SEM) imaging was carried out using a JEOL JSM-7610F SEM (JEOL LTD, Japan).

Neutrophils (1 × 10^6^) were added to 46 h biofilms grown on coverslips in 24-well plates and incubated for another 2 h at 37°C in a 5% CO_2_ atmosphere (condition 3). Control biofilms without neutrophils were included in the experimental design. Coverslips were removed carefully from the wells using micro-tweezers, transferred to a fresh well and rinsed once in PBS. Biofilms were fixed in 4% PFA for 10 min at room temperature [25] and then washed with PBS. For live/dead imaging of biofilms, FilmTracer™ LIVE/DEAD™ Biofilm Viability Kit (Invitrogen, Renfrew, UK) was used according to the manufacturer’s instructions. After 20 min, the coverslips were mounted onto glass slides with a glycerol-based mounting medium (SlowFade Gold Antifade Mountant, Thermo Scientific, Basingstoke, UK) and immediately imaged. The Biofilm Viability Kit is primarily designed for assessing biofilm viability but also targets eukaryotic cells such as neutrophils, as the kit contains SYTO 9 and PI, both DNA-intercalating dyes not specific to bacterial DNA. These have also been used in the literature to assess neutrophil viability [26,27].

Imaging was conducted using a 40× oil immersion objective (Zeiss Objective EC Plan-Neofluar 40X/1.30 Oil DIC M27, FWD = 0.21 mm). The two stains were first imaged separately to control for any cross-bleed between channels. The excitation used was 488 nm for SYTO®9 and 561 nm for propidium iodide (PI). Z-stack images were acquired immediately after staining with a 20× objective (Zeiss Objective EC Plan-Neofluar 20X/0.5, WD = 2.0 mm). Typically, dead or damaged cells display both green fluorescence from SYTO®9 and red fluorescence from PI, indicating loss of membrane integrity. The percentage of neutrophil viability within the sample was calculated by the rate of neutrophils of red/yellow colour versus green cells with a diameter of 9–15 µm. Four different images obtained from each corner of the biofilm were assessed by manual cell counting. To determine neutrophil ability to attach to *S. oralis* biofilms, the supernatant of the biofilms was collected (*n* = 3 in triplicate), stained with trypan blue, and viable cells were counted.

SEM imaging of biofilms (condition 3) was performed by transferring the biofilm into a well containing 1 ml of paraformaldehyde fixative for 20 min, followed by a primary fixative of 2.5% glutaraldehyde (019095, Polysciences, PA, USA) for 1 h. Samples were washed three times with PBS for 5 min each. Biofilms were then dried with ascending concentrations of ethanol (20% to 100%) for 15 min each. Finally, the samples were air-dried, then the coverslips containing the biofilm were placed onto 25 mm aluminium stubs (G3024 Agar Scientific) with carbon conductive tape and coated in gold for 90 sec (Denton Vacuum Desk II). The biofilms were examined at an accelerating voltage of 15 kilovolts (kV).

## Biofilm quantity assessment by crystal violet (CV) staining

After 48 h of *S. oralis* biofilm development (conditions 1–3), coverslips containing biofilm as well as negative control coverslips containing only neutrophils were transferred to a new 24-well plate. Non-adherent cells were removed by washing once with ultrapure water (Milli-Q, Merck Millipore, Carrigtwohill, Ireland) and air-dried for 1 h at RT. After complete drying of biofilms, 1 ml of 0.1% CV solution was added to each well for 30 min. The CV dye was then discarded, and adherent biofilm was washed three times with ultrapure water. Coverslips were air-dried at room temperature, followed by adding 1 ml of 30% acetic acid and incubation for 15 min [28]. The CV-acetic acid solution was then transferred into a clear 96-well plate (200 µl) and absorbance was measured in a plate reader (ELx800, BioTek) at 600 nm. In order to address any neutrophil uptake of the CV stain, the readings from control wells containing only neutrophils (1 ml of 1 × 10^6^ cells) were subtracted from the absorbance readings of wells containing both biofilm and neutrophils.

## Biofilm surface adhesion measurement

Neutrophils (1 × 10^6^) in TSB were added to 46 h *S. oralis* biofilms and incubated for another 2 h at 37°C in 5% CO_2_ (condition 3). Biofilms without neutrophil addition were used as controls. Next, coverslips were transferred into a fresh 24-well plate and washed with 1 ml ultrapure water, followed by addition of 1 ml of ultrapure water. Biofilm adhesive strength was assessed by transferring the 24-well plate to an orbital plate shaker (Thermo Scientific, Basingstoke, UK) with a 25 mm amplitude and agitating at 550 rpm/min for 10 min. Water in each well was collected and replaced every minute, and collected samples were vortexed. Next, absorbance at OD_600_ was measured using a spectrophotometer. After 10 min, the remaining biofilm was resuspended in 1 ml water and removed mechanically from the coverslip by scraping and vortexing, followed by spectrophotometry. The measured absorbance in the final sample was directly proportional to non-detached biofilm, whereas that of all other samples corresponded with detached biofilm.

## ROS quantification

ROS production of neutrophils in response to *S. oralis* biofilms was determined using chemiluminescence [13,29] under condition 3. White flat bottom non-treated 96-well plates (Corning 3912, Fisher Scientific, Loughborough, UK) were blocked with 1% BSA in PBS overnight at 4°C and washed with PBS prior to use. Each well was inoculated with 200 µl per well of bacterial suspension in TSB grown overnight and incubated for 46 h (37°C, 5% CO_2_) to form biofilms.

Next, the supernatants were removed, and biofilms were washed once with 200 µl PBS. 100 μl of 1 × 10^5^ freshly isolated neutrophils in gPBS (PBS supplemented with glucose and cations [7.75 g NaCl, 0.2 g KH_2_PO_4_, 1.5 g K_2_HPO_4_, 1.8 g glucose, 0.15 g CaCl_2_, 143 mg MgCl_2_; all reagents were obtained from Merck, Germany]) were added to each well. GPBS was added to negative control wells not containing biofilms, and 50 nM phorbol 12-myristate 13-acetate (PMA; Merck, Germany) was added to positive control wells.

Luminol (3 mm) was used to quantify total and extracellular ROS. Isoluminol (3 mm) was used to quantify intracellular and extracellular ROS. Luminol and isoluminol were purchased from Merck. For intracellular ROS 15,000 units/ml of superoxide dismutase (SOD) (Merck, S9697-15KU) and 2000units/ml of catalase (Merck, E3289) were added to each well-containing luminol. For extracellular ROS, 1.5 units/µl of horseradish peroxidase (HRP) (Merck, P8415-5KU) was added to isoluminol-containing samples. Luminescence was measured in a luminometer (Berthold Tristar2; Berthold Technologies, Harpenden, UK, with MikroWin2000 software; Informer Technologies, Madrid, Spain). Luminescence was recorded every 3 min over a time period of 120 min at 37°C. All readings were expressed as relative light units (RLUs).

## Quantification of eDNA and NETs

For the quantification of extracellular DNA (eDNA) under condition 3, non-treated clear 96-well plates (Corning 3370) were blocked with 1% BSA in PBS overnight at 4°C and washed with PBS prior to use. Each well was inoculated with 200 µl per well of *S. oralis* suspension in TSB and incubated for 46 h (37°C, 5% CO_2_) to form biofilms. Subsequently, the supernatants were removed, and biofilms were washed once with 200 µl PBS. Next, 200 µl of RPMI 1640 containing 1 × 10^5^ isolated neutrophils were added to each well. 0.2% hypochlorous acid (HOCl) was used for positive controls to induce NET formation in neutrophils, as previously described [13]. Subsequent to stimulation, the plate was covered and incubated for 2 h (37°C, 5% CO_2_). Post incubation, a final well concentration of 1 unit/ml of micrococcal nuclease (MNase; Invitrogen, Renfrew, UK) in PBS was added to each well [13].

The culture plate was incubated at 37°C for 10 min, leading to digestion of NETs in order to detach these from neutrophils. The plates were centrifuged for 10 min at 1,800 × g. Next, 150 µl of the supernatant containing all digested extracellular DNA, was carefully transferred, without disturbing the pellet, to the wells of a black polystyrene plate, and 15 µl of 10 µM SYTOX™ Green nucleic acid stain (Invitrogen, Renfrew, UK) were added to each well. Fluorescence was recorded as arbitrary fluorescent units (AFU) in a mmicroplate reader (Spark®, Tecan; software SparkControl, v. 2.3, Tecan) at excitation of 485 nm and emission of 525 nm. Of note, this assay quantifies both bacterial and mammalian extracellular DNA. Spontaneous extracellular DNA generation from wells containing unstimulated neutrophils was subtracted from wells containing both neutrophils and biofilms in order to assess eDNA levels.

## Deoxyribonuclease (DNase) production by S. oralis

Bacterial ability to produce DNase was assessed by culturing bacteria on DNase media (Oxoid, Basingstoke, UK). DNase media was prepared according to the manufacturer’s instructions. Two to three bacterial colonies from overnight TSA culture were inoculated onto a 1.3 × 1.3 cm area of DNase agar for 3 days at 37°C in a 5% CO2 atmosphere. After incubation, the plate was flooded with 1N hydrochloric acid (HCl; VWR, Radnor, PA, US) and positive DNase activity was visualised as clear zones surrounding the bacterial growth area. *Staphylococcus aureus* (ATCC 6571) cultures were used as a positive control.

## Analysis of bacterial DNA uptake by neutrophils

Bacterial DNA was extracted to test the biofilm eDNA interaction with neutrophils. Bacteria were cultured in TSB overnight in a shaking incubator and DNA was isolated using the QIAamp® DNA extraction mini kit (Qiagen, Germantown, Maryland). DNA extraction was carried out according to the manufacturer’s instructions, and the DNA concentration was measured using a Qubit 2.0 Fluorometer (Invitrogen, Singapore). Neutrophils were isolated and adjusted to 1 × 10^6^ cells/ml in gPBS.

The neutrophil suspension was then stained with 1 µg/ml Hoechst 33258 for 20 min in the dark, and subsequently washed twice in gPBS and centrifuged at 1600 rcf for 2 min. Isolated *S. oralis* DNA was stained with 3 µg/ml PI for 20 min. DNA was washed twice with PBS and centrifuged for 2 min at 1600 rcf. 100 µl of stained neutrophils were added to 100 µl of DNA and incubated at 37C° on a shaking rack. The mixture was transferred to round bottom polystyrene FACS tubes and analysed using FACS Canto II (BD Biosciences) with a medium flow rate (event acquisition rate of 10,000 events/sec). Flow cytometry data were analysed using the associated FACSDiva software (BD Biosciences). The neutrophil gate was determined by forward (FSC) and side scattering (SSC). Part of the mixture described above (5 µl) was transferred to a glass slide and examined microscopically (Zeiss Axiovert inverted 200 M fluorescence microscope).

## Assessment of viable bacterial cells in biofilms

Media was removed from all biofilm-containing wells of a 24-well plate after 48 h (condition 3). Biofilms were washed once with 1 ml PBS. Next, 1 mL of PBS solution was added to each well, and biofilms were fully resuspended by vigorous pipetting. 100 μL of the suspension was transferred to a new 24-well plate, where 10-fold serial dilutions were prepared in 900 μL PBS until a 100,000 × dilution was achieved. 100 μL of each sample was plated onto TSA using five sterile plating beads (Merck, Germany) per agar plate. CFUs were enumerated after 24 h of incubation at 37°C. Complete biofilm detachment from the first 24-well plate was confirmed by CV staining of these wells after transferral of the biofilm suspension.

## Statistical analysis

All statistical analyses were performed in GraphPad Prism 9 software (San Diego, CA, USA). A p-value of ≤ 0.05 was considered statistically significant. Normality was assessed using the Shapiro – Wilk test. For parametric data, t-testing, one-way or two-way ANOVA was applied. For non-parametric data, Kruskal-Wallis test was used. All parametric data are presented as mean values ± standard deviations, while non-parametric data are shown as medians and ranges.

## Results

### Neutrophils decrease biofilm quantity transiently

**Figure 1.**
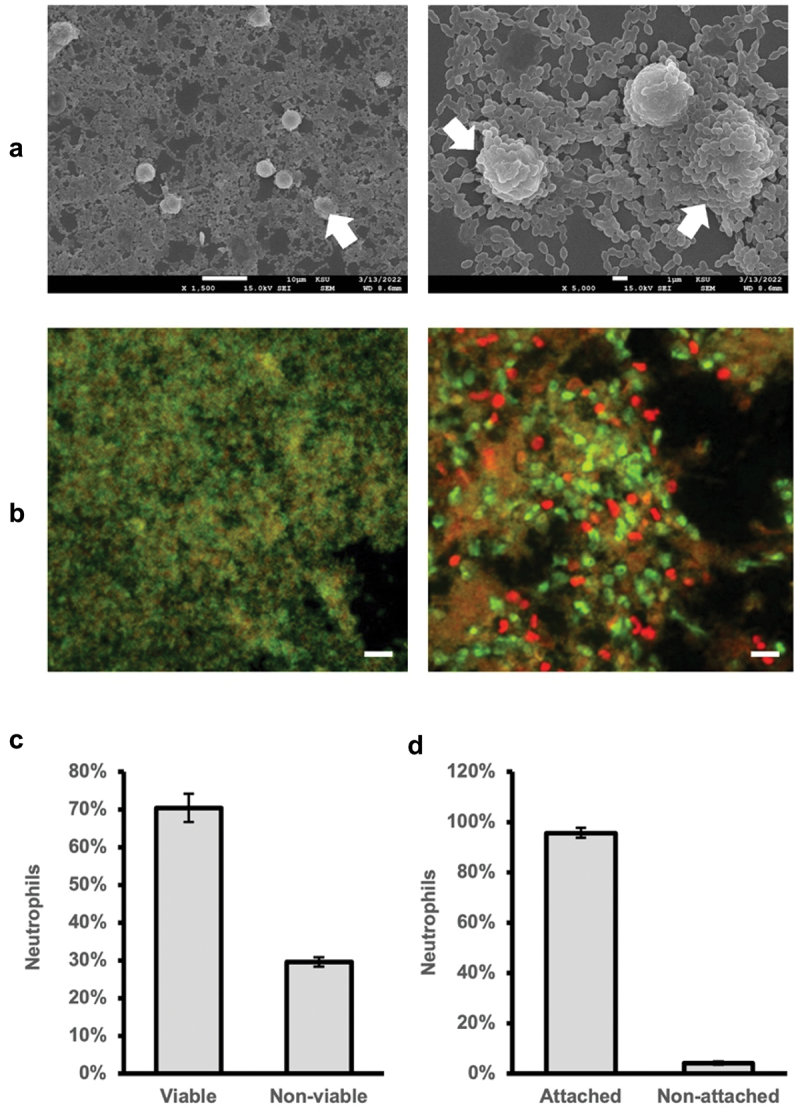
Presence of neutrophils within biofilms. a) SEM images of *S.Oralis* biofilms after neutrophils were added and incubated for 2 h, showing neutrophils are retained within these biofilms and become overlaid with bacteria rapidly (arrows). 1,500× (left, scale bar: 20 μm) and 5,000× (right, scale bar: 5 μm) magnification. b) Representative fluorescence images of biofilms without (left) and with (right) neutrophils. Merged images from PI and Syto9 staining at 40× magnification (scale bars: 20 μm). Live (Syto9, green) and dead (PI, red) neutrophils are seen. c) percentage of viable vs. non-viable neutrophils as assessed by counting green vs. red/yellow stained cells (*n* = 3 biological repeats in quadruplicate). d) percentage of neutrophils adherent vs. not adherent to biofilms in multiwell plates, assessed by cell counting in biofilm supernatants (*n* = 3 in triplicate).

In our experimental setup, neutrophils were successfully co-cultured with biofilms, and were found to be embedded within the biofilm structure, with neutrophils being overlayed with bacterial layers (Figure 1a, b). Both live and dead neutrophils were observed microscopically by live/dead staining. After interacting with *S. oralis* biofilm, neutrophils were found to exhibit a decrease in their viability by one third (mean decrease: 29.6%, SD: 1.25%; Figure 1c), as demonstrated by cell counting following LIVE/DEAD imaging.

Neutrophils showed good survival (80%) in TSB with the least variability (SD of 28%) after 4 h, compared with their survival in RPMI-1640 + 10% FBS (**Suppl. Fig. S2A**). In addition, TSB supported biofilm growth significantly better than SB and RPMI (**Suppl. Fig. S2B**). Consequently, TSB was used in all downstream studies. In addition, we found only a relatively low proportion (mean: 4.3%, SD: 0.65%; Figure 1d) of all added neutrophils in the supernatant after the first media change, indicating a high retention of neutrophils within the biofilms.

**Figure 2.**
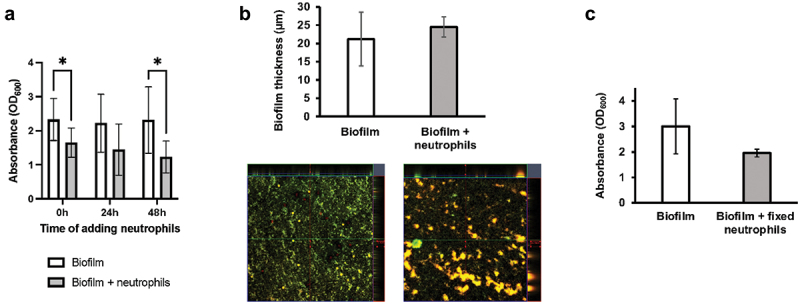
Effect of neutrophils on biofilm quantity. a) *S. oralis* 48 h biofilm quantity with neutrophils added before biofilm development (0 h), after 24 h or after 46 h of biofilm development, quantified by CV technique (*n* = 9 in triplicate wells). Mean values ± SD are shown, two-way ANOVA with šídák‘s multiple comparison test,*p ≤ 0.016. b) quantification (bar graph) of biofilm thickness from Z-stack CLSM images (lower panel, 20× magnification) with representative colour-merged images (SYTO9 and PI). *S. oralis* 48 h biofilm quantity with neutrophils added for 2 h. *N* = 3 donors in duplicate (two Z-stack images were taken from different areas of the biofilm). Mean values ± SD, two-tailed t-test, no significant difference was detected. c) *S. oralis* 48 h biofilm quantity with PFA-fixed neutrophils added for the final 2 h. Fixed neutrophil-only readings were subtracted from biofilm + fixed neutrophils readings. Mean values ± SD are shown, two-tailed t-test, *p* = 0.053, *n* = 6 in triplicate wells.

Neutrophils added to 46 h old biofilms for 2 h, and neutrophils added before initiation of biofilms led to a significant decrease in biofilm quantity as determined by CV technique (Figure 2a). The biofilm decrease detected in samples with neutrophils added after 24 h, however, was not significant. These decreasing trends were not confirmed by CLSM, where the maximum thickness of the biofilms was estimated from z-stack horizontal images (Figure 2b). Notably, non-viable neutrophils had a similar effect as live neutrophils on the biofilm quantity, although this was not statistically significant (Figure 2c).

### Biofilm surface adhesion was unaffected by neutrophils

**Figure 3.**
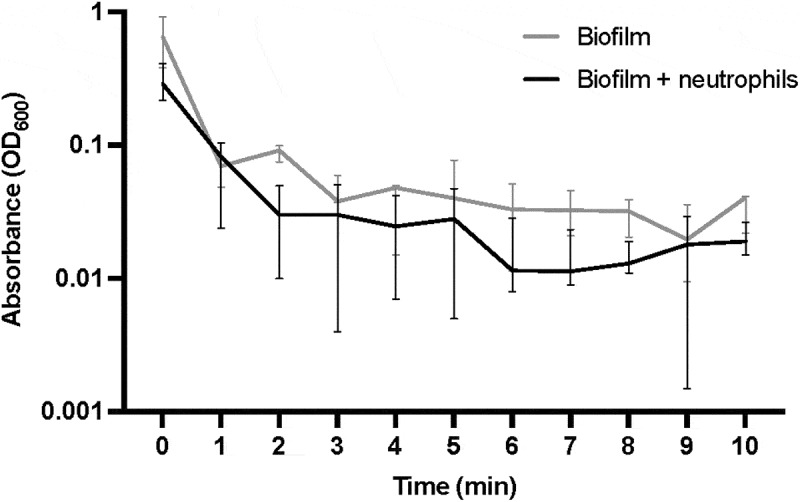
Effect of neutrophils on *S. oralis* biofilm adhesion. Neutrophils were added to 46 h old biofilms for 2 h before determining adhesive strength, biofilm detachment from glass surfaces over time is shown as median values and interquartile range. *N* = 7 in triplicate wells, Kruskal-Wallis test with Holm-šídák post hoc test, no significant differences in attachment between biofilms and time points were observed.

The effect of neutrophils on *S. oralis* biofilm surface adhesion was analysed by applying shear forces. Time point zero provides the sum of all biofilm detachment and remaining biofilm on the coverslips. *S. oralis* biofilm detachment was not significantly different from those biofilms containing neutrophils over the time course of 10 min (Figure 3), suggesting that neutrophils may not influence biofilm adhesive strength.

### Neutrophils produce intracellular ROS in response to biofilms

**Figure 4.**
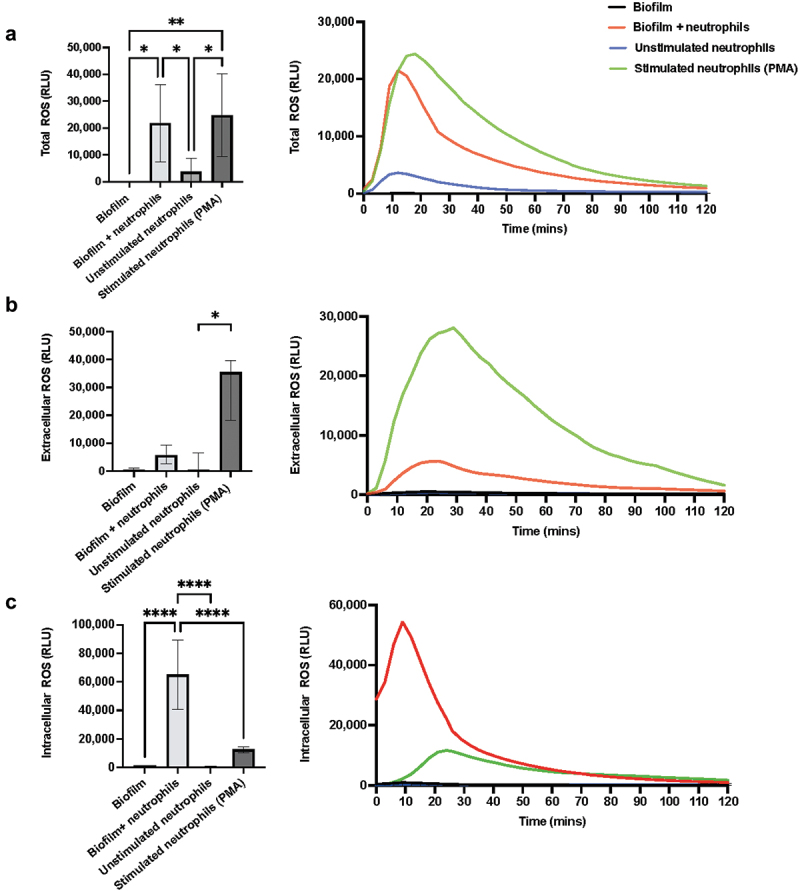
Neutrophil ROS release in response to   46 h old *S. oralis* biofilms. Chemiluminescence was measured over a time course of 120 min, shown as relative light units (RLU). 50 nM PMA was used as the positive control, and unstimulated neutrophils as the negative control. *N* = 6 in triplicate wells. a) total ROS (mean values ± SD; one-way ANOVA with Tukey’s multiple comparisons test *p ≤ 0.04, **p = 0.004), b) extracellular ROS (median values ± interquartile ranges; Kruskal-Wallis test with Dunn’s multiple comparisons test, *p = 0.05) and c) intracellular ROS production, (mean values ± SD; one-way ANOVA with Tukey’s multiple comparisons test ****p < 0.0001).

Significantly increased total ROS (Figure 4a) was observed in neutrophils stimulated with 46 h old *S. oralis* biofilms for 2 h, relative to the negative control (unstimulated neutrophils) and was equivalent to the positive (PMA) control. Whilst extracellular ROS release was not increased in these samples (Figure 4b), intracellular ROS showed levels significantly higher than both the negative and positive controls (Figure 4c). Generally, peak ROS release in response to the biofilm stimulus occurred within the first 20 min, followed by a slow decrease to baseline levels over the course of 2 h. *S. oralis* biofilms alone did not produce significant amounts of ROS.

### Neutrophils decrease bacterial eDNA but do not produce NETs in response to biofilms

**Figure 5.**
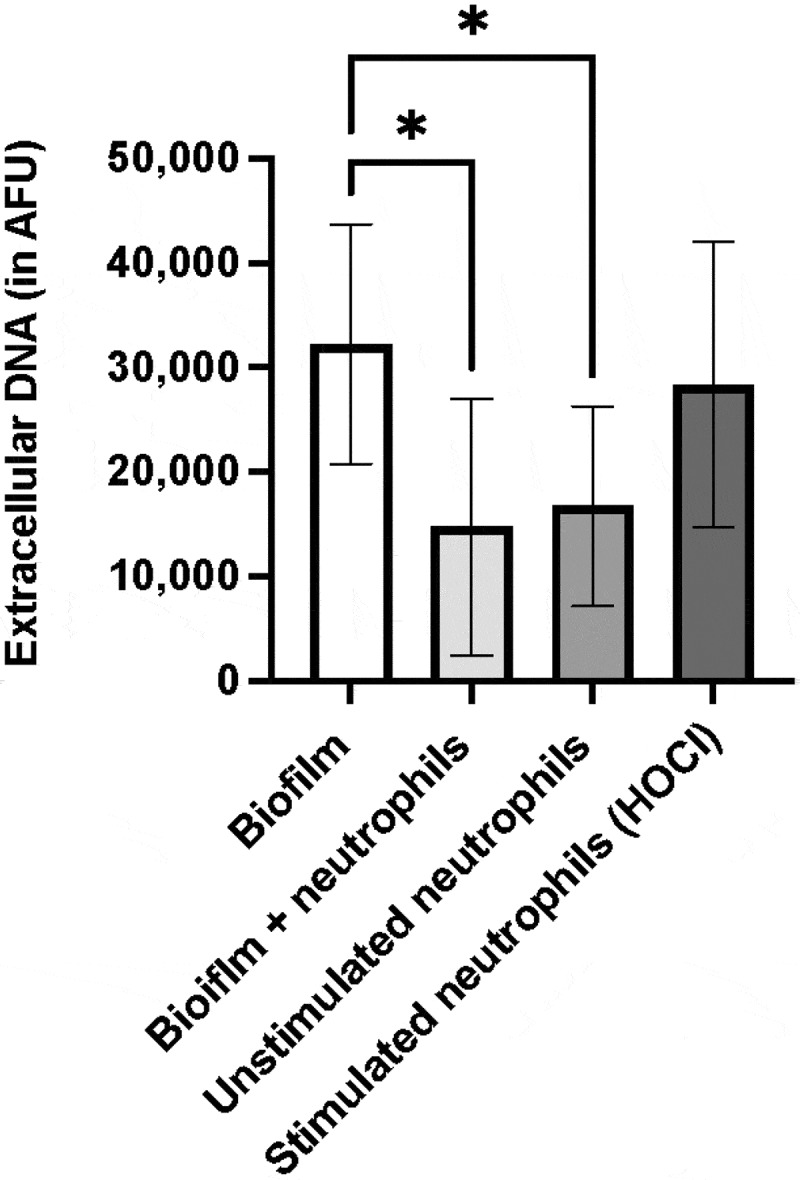
Extracellular DNA quantification in 48 h old biofilms and neutrophils. This assay quantifies both bacterial eDNA and neutrophil extracellular DNA, indicating NET release (*n* = 9 in triplicate wells). Mean values ± SD are shown, one-way ANOVA with Tukey‘s multiple comparison test,*p ≤ 0.04. Mean value of unstimulated neutrophils was subtracted from that of biofilm + neutrophils. AFU = arbitrary fluorescent units, HOCl = hypochlorous acid (positive control).

Several bacteria are known to produce DNAses that can degrade NETs and thus help avoid entrapment and/or killing by NETs [30]. *S.oralis* did not produce DNase enzyme after 72 h of incubation as compared with *S. aureus* which is known to produce high levels of DNAse [31] (**Suppl. Fig. S3**). In our fluorescence-based DNA-detection assay, bacterial eDNA was observed in biofilm samples (Figure 5). Interestingly, these eDNA levels significantly decreased in samples containing both biofilms and neutrophils, indicating a reduction of biofilm eDNA mediated by neutrophils. In addition, neutrophils were not found to produce NETs in response to *S. oralis* biofilms in this assay, compared with the negative control (unstimulated neutrophils).

### Bacterial DNA is bound by neutrophil surfaces

**Figure 6.**
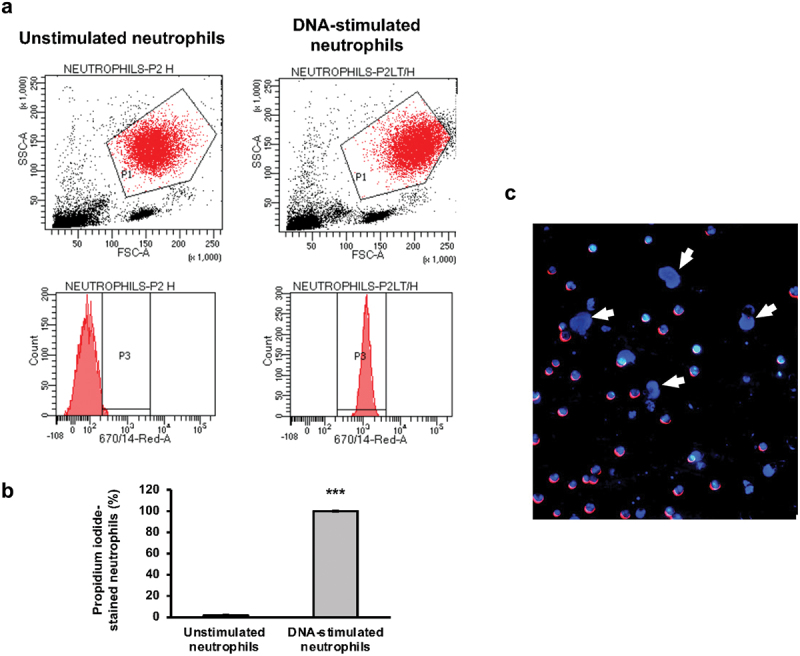
Neutrophil interaction with *S. oralis* isolated DNA. a) Representative flow cytometry scatter plots and histograms for untreated (left) and *S. oralis* dna-treated (right) neutrophils. Neutrophil gating was based on forward (FSC) and side scatter (SSC) as well as on Hoechst 33258 staining. A neutrophil population shift towards the right is seen in the stimulated sample. **B**) Quantification of the percentage of neutrophils staining positively for propidium iodide (PI), indicating co-localisation with bacterial DNA. Mean values ± SD are shown, one-tailed paired t-test, ***p < 0.0001. **C**) Neutrophils pre-stained with Hoechst 33258 (blue) and incubated with isolated and pre-stained *S. oralis* DNA (PI, red); 20× magnification, scale bar represents 20 μm. Localisation of *S. oralis*DNA on neutrophil surfaces as well as some dead/dying cells (white arrows) are seen.

Neutrophils’ ability to uptake extracted bacterial DNA was investigated to better understand the decrease in biofilm eDNA detected in this study. Flow cytometry assessment showed neutrophil-bacterial DNA co-localisation in ≥ 98.3% of the cell population compared to ≤ 1.9% in unstimulated neutrophils. Furthermore, a higher forward scatter in stimulated neutrophils indicated an increase in cell size (figure 6a,b). Fluorescent imaging also showed DNA localisation in the neutrophil membrane areas (Figure 6c).

### Neutrophils increase CFUs within biofilms

**Figure 7.**
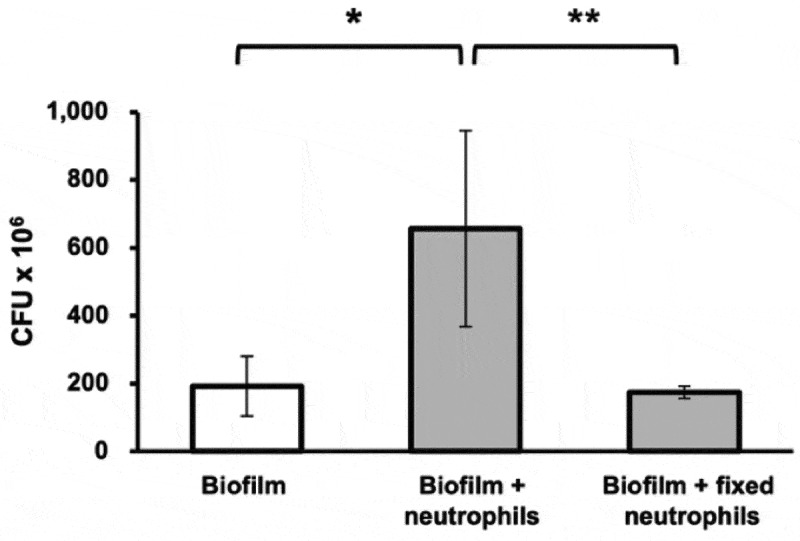
48 h *S. oralis* biofilm CFUs with and without live and non-viable (fixed) neutrophils.

The presence of viable neutrophils in biofilms significantly increased CFUs, whilst non-viable (fixed) neutrophils did not have the same effect (Figure 7). Of note, this increase in CFU was seen after incubation of the biofilms with neutrophils for 2 h.

Mean values ± SD are shown, one-way ANOVA, *p < 0.05, **p < 0.01 (*n* = 6 in triplicate).

## Discussion

Neutrophils play a key role in innate immune defence against oral biofilms. *In vivo*, NETs and biofilms are inextricably linked [32]. *S. oralis* was selected in this study because it is an essential early coloniser in the oral cavity, allowing other oral bacteria to attach and the biofilm to mature [33]. To the best of our knowledge, no research has been carried out to date to investigate the effects of neutrophils on *S. oralis* biofilms. Our findings indicate that neutrophils are well-retained in an *in vitro* co-culture assay system, being consistent with previous reports [34].

The presence of neutrophils before biofilm development had no significant impact on the attachment stage of *S. oralis*, however, a short-lasting neutrophil-mediated biofilm quantity decrease was observed. The lack of a longer term anti-biofilm impact by neutrophils may be explained by bacterial growth overcoming the neutrophil-mediated antimicrobial effects of neutrophils. Indeed, *S. oralis* doubling time *in vitro* is approximately 1 h [35,36], whilst neutrophils have an estimated life span of up to 20 h *in vitro* after isolation [37]. Furthermore, in the oral cavity, approximately 30,000 neutrophils enter the gingival crevice per minute [38], hence, antimicrobial effects are sustained *in vivo* but this may not necessarily be the case *in vitro*. In support of our finding, biofilm quantity was not significantly affected by non-viable neutrophils, although the same trend as in live neutrophil samples was seen. This results suggests that neutrophils may not require to be viable in order to decrease biofilm quantity.

The significant but overall low anti-biofilm effects of neutrophils shown by CV biofilm quantity quantification could also be due to the finding that the majority of adhered neutrophils were found to be covered by a bacterial layer in our SEM images. This bacterial wall may have acted as a protective barrier by neutralising toxic components produced by neutrophils [39]. Furthermore, due to the high density of bacteria and the protective nature of the biofilm’s EPS, neutrophils are likely to have a decreased phagocytic function [40]. In contrast to our CV quantification results, Z-stack confocal imaging showed no significant difference in biofilm thickness. This is likely attributable to neutrophils contributing to the biofilm quantity as assessed microscopically. In future experiments, differential staining techniques could enable the computational separation of neutrophils and biofilms in order to determine the true thickness of biofilms.

Although *S. oralis* biofilm has been documented to induce NET formation [18,41], we were not able to quantify NETs in response to our *S. oralis* biofilms. Bacterial DNases did not cause the decrease in NETs as our *S. oralis* strain was confirmed to be DNase-negative, and to the best of our knowledge, no endo- or exonucleases are released extracellularly by neutrophils. The absence of NET detection in our study may be attributable to the fact that *S.oralis* is considered a periodontal health-associated commensal, a yellow Socransky complex species, with no or low pathogenicity [42]. Similarly, our previously published work exploring NET release in response to a closely related *S. oralis* strain under planktonic conditions also showed no NET formation [13].

On the other hand, we found eDNA in *S. oralis* biofilms, which is likely to be produced as part of the biofilm EPS, providing structural stability [4]. Unexpectedly, *S. oralis* biofilm eDNA decreased after the addition of neutrophils, which raises the hypothesis that eDNA may be bound or taken up by neutrophils. This hypothesis was tested by fluorescently investigating the co-localisation of neutrophils and isolated bacterial DNA, the latter of which was found to be adjacent to neutrophil cell surfaces, indicating possible DNA binding to the cell membrane. Additionally, flow cytometry analysis indicated increased forward scatter in the neutrophil population, indicating an enlargement in cell size. Microscopically, neutrophils not showing DNA co-localisation displayed enlargement, indicating necrosis [43].

Neutrophils are known to increase in size when activated *in vitro* [44,45] and this can be observed by flow cytometry by a higher forward scatter [46]. Our findings suggest that the neutrophil size transition was caused by bacterial DNA. Endosomal Toll-like receptor 9 (TLR9) in human neutrophils recognises unmethylated-CpG motifs in bacterial DNA [47]. The receptor is understood to exist intracellularly only and to be activated upon DNA internalisation [48]. However, there is now evidence that TLR-9 could be located on the surface of neutrophils and sense bacterial DNA via an endocytosis-free process leading to neutrophil activation [49]. Hence, if *S. oralis* DNA was indeed bound to outer neutrophil membranes, it is possible that cell activation could have been mediated by TLR-9. However, the precise methods by which neutrophils recognise and bind to microbial eDNA requires further investigation using mechanistic studies and higher resolution microscopy.

Furthermore, activation of neutrophils was confirmed by increased total and intracellular ROS. An increase in intracellular ROS in response to bacterial stimulation indicates phagocytotic activity [50]. In addition, intracellular ROS can be also formed in the absence of phagosome formation [51]. Compared with a previous study by our group utilising planktonic *S. oralis*, biofilm-stimulated neutrophils produced higher total ROS than neutrophils stimulated with planktonic bacteria, whilst there was no difference in extracellular ROS production between biofilm and planktonic bacteria [13]. These data suggest that neutrophils may respond differently to *S. oralis* biofilms than to the planktonic form of the bacteria. The increase in total ROS may be due to the higher bacterial numbers in biofilms or to biofilm-produced stimulatory agents such as EPS [52,53]. Overall, the increase in mainly intracellular ROS indicates no extracellular release of toxic ROS, and, therefore, lower potential for collateral host tissue damage. Our results support the findings of Oveisi et al., who reported increased intracellular ROS in response to *S. oralis* monospecies biofilms [41].

eDNA is vital in biofilm maturation and has been found in various bacterial species to be important for intercellular connections within biofilm, contributing to its three-dimensional structure [54]. As a result, its absence leads to unstable biofilms prone to detachment and collapse of the matrix. However, in our study of neutrophils, the decrease in eDNA had no effect on biofilm detachment, indicating that other mechanisms are more important in biofilm adhesion, such as antigen I/II family proteins, lipoproteins, pili, fimbriae, as well as other adhesins [55,56]. To strengthen the results obtained through our approach of assessing biofilm surface adhesion, complementary methods, such as microscopy, should be applied in future studies. Whilst some approaches have been described in the literature to quantify bacterial adhesion or detachment by applying shear stress through flow or shaking [57,58], there is currently no widely used method, and none had previously been described for *S. oralis* biofilms.

The number of viable bacterial cells unexpectedly increased within the biofilm after neutrophil contact for 2 h. Similarly, *Pseudomonas aeruginosa* biofilm enhancement by dead neutrophils has been reported previously [59], however, only biofilm quantity, not CFUs were assessed in this study. Other studies have reported that bacteria can use dead leukocytes as a proliferation niche [60] and that a process termed death-induced nutrient release (DINNR) in the context of colonic cell apoptosis can enhance bacterial growth [61]. A possible explanation in our case is an increased transport of small nutrient molecules and faster removal of waste products within biofilms after its partial disruption [62].

Indeed, Melaugh et al. reported that in biofilms, cells outside of bacterial aggregates grow more rapidly than those in central aggregation regions, as they have better access to nutrients [63]. Moreover, the authors report that bacterial growth is highly heterogenous across a biofilm, depending on the surface area exposed to nutrients. Hence, it is possible that through neutrophil-mediated biofilm disruption, such as through loss of extracellular matrix and eDNA, the biofilm surface is enlarged with better access to nutrients. This theory is in support of our data showing no CFU increase after exposure to fixed neutrophils, as these neutrophils may have been unable to bind eDNA, to actively disturb the biofilm and may also not have been a suitable nutrient source due to chemical fixation. The rapid bacterial growth within 2 h is plausible in light of the fact that bacterial doubling time during the exponential phase is approximately 1 h [35,36].

As a limitation of the study, quantification of eDNA and measurement of its reduction, especially in biofilm-host cell co-cultures, where non-membrane-permeable fluorescent DNA dyes are used, is difficult to achieve. This is because bacterial eDNA cannot easily be distinguished from dead bacterial cells and dead or damaged host cells or NETs. However, approaches like fluorescent in-situ hybridisation (FISH) in combination with immunolabeling of DNA may improve differential assessment of different types of DNA in the same sample [4]. This method would also allow for better assessment of biofilm quantity in mixed samples where neutrophils are also present.

## Conclusions

Although neutrophil defences against oral biofilms have been previously investigated, their complex interactions are not well understood. Translating our findings to *in vivo* conditions requires consideration of additional aspects such as a multitude of other host responses, more complex multispecies biofilms and local environmental factors. However, our findings reveal an anti-biofilm property of neutrophils in the context of *S. oralis*, which is short-lasting, and may be related to an uptake or binding of eDNA by neutrophils without affecting biofilm surface adhesion and bacterial CFUs. Conversely, this commensal biofilm activated mainly intracellular ROS but elicited no NET formation. Future studies should employ multispecies biofilms also involving more pathogenic species, and an *in vitro* system in which neutrophils are replenished. Furthermore, the possible eDNA-diminishing property of neutrophils warrants further investigation, as further insights into the effects of eDNA reduction could form the basis for pharmaceutical targeting of oral biofilms.

## Supplementary Material

Supplemental Material

## Data Availability

All relevant data are provided within this paper, further details are available upon request.

## Key messages

Neutrophils are able to reduce *in vitro S. oralis* biofilm quantity, however, this effect is relatively short-lasting. A possible mechanism is the reduction of biofilm eDNA by neutrophils. Towards this commensal species biofilm, neutrophils respond with increased intracellular ROS but not with extracellular ROS or NET release. At the same time, dying neutrophils may serve as a nutrient reservoir for bacteria.
