# Supplementary material for: Bidirectional effects of neutrophils on Streptococcus oralis biofilms in vitro
Source: J Oral Microbiol. 2025 Jan 23;17(1):2453986. doi: 10.1080/20002297.2025.2453986 (PMC11758797; doi:10.1080/20002297.2025.2453986)
Supplement: Supplemental Material [file ZJOM_A_2453986_SM8927.docx]

**Supplementary Figure 1**

**
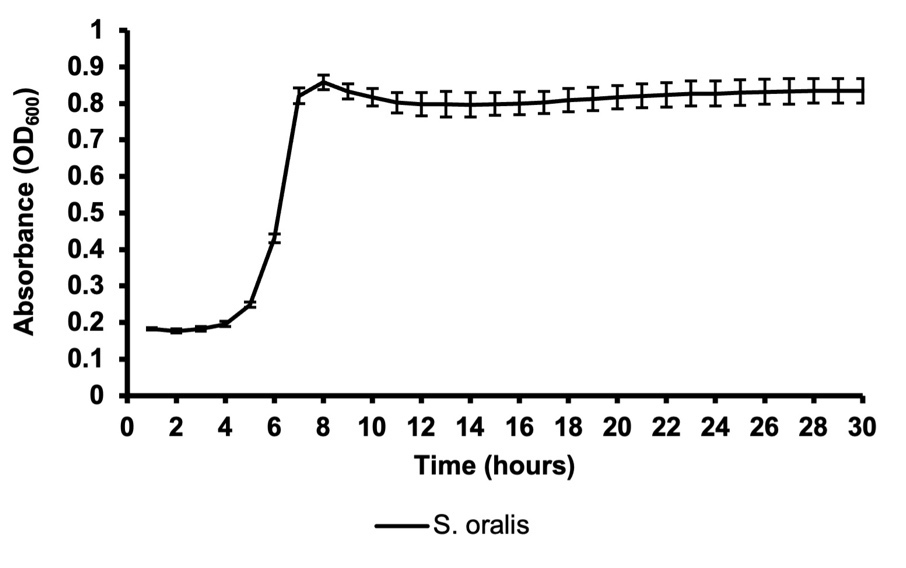
**

**Suppl. Figure 1. Growth curve for planktonic S. oralis in TSB**.

Readings were obtained every hour for 30 h in a plate reader and are displayed as mean values ± SD. Plates were incubated at 37°C (n=3 independent experiments).

**Supplementary Figure 2**


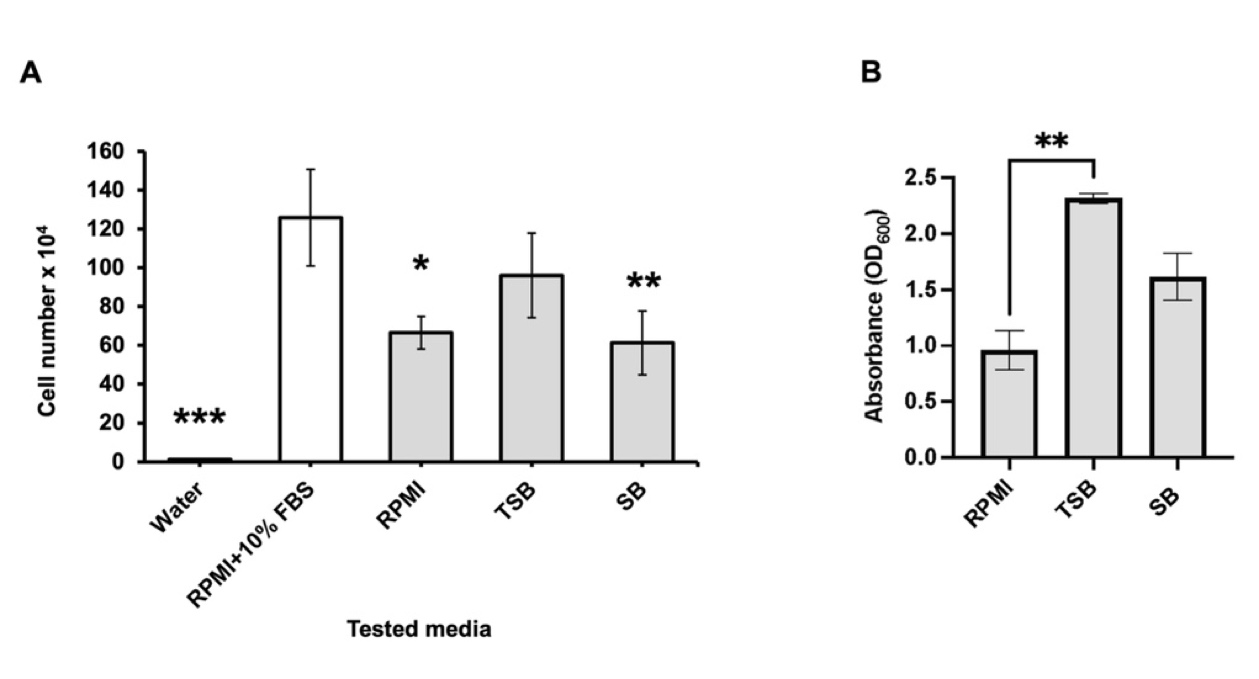


**Suppl. Figure 2**. **Determination of the optimum media for co-incubation of biofilms and neutrophils. A**) Live neutrophil numbers in different growth media after 4h of incubation. RPMI+10% FBS was used as a positive control for neutrophil survival. Repeated measures one-way ANOVA; ***p<0.0001, **p≤0.01, *p≤0.05 compared to the positive control. Mean values ± SD are shown, n=6 in triplicate. **B**) *S. oralis* biofilm growth in different media assessed by CV technique. Mean values ± SD are shown, repeated measures one-way ANOVA with Tukey’s post hoc test, **p<0.01. RPMI: RPMI 1640, TSB: tryptic soy broth, SB: Schaedler broth.

**Supplementary Figure 3**


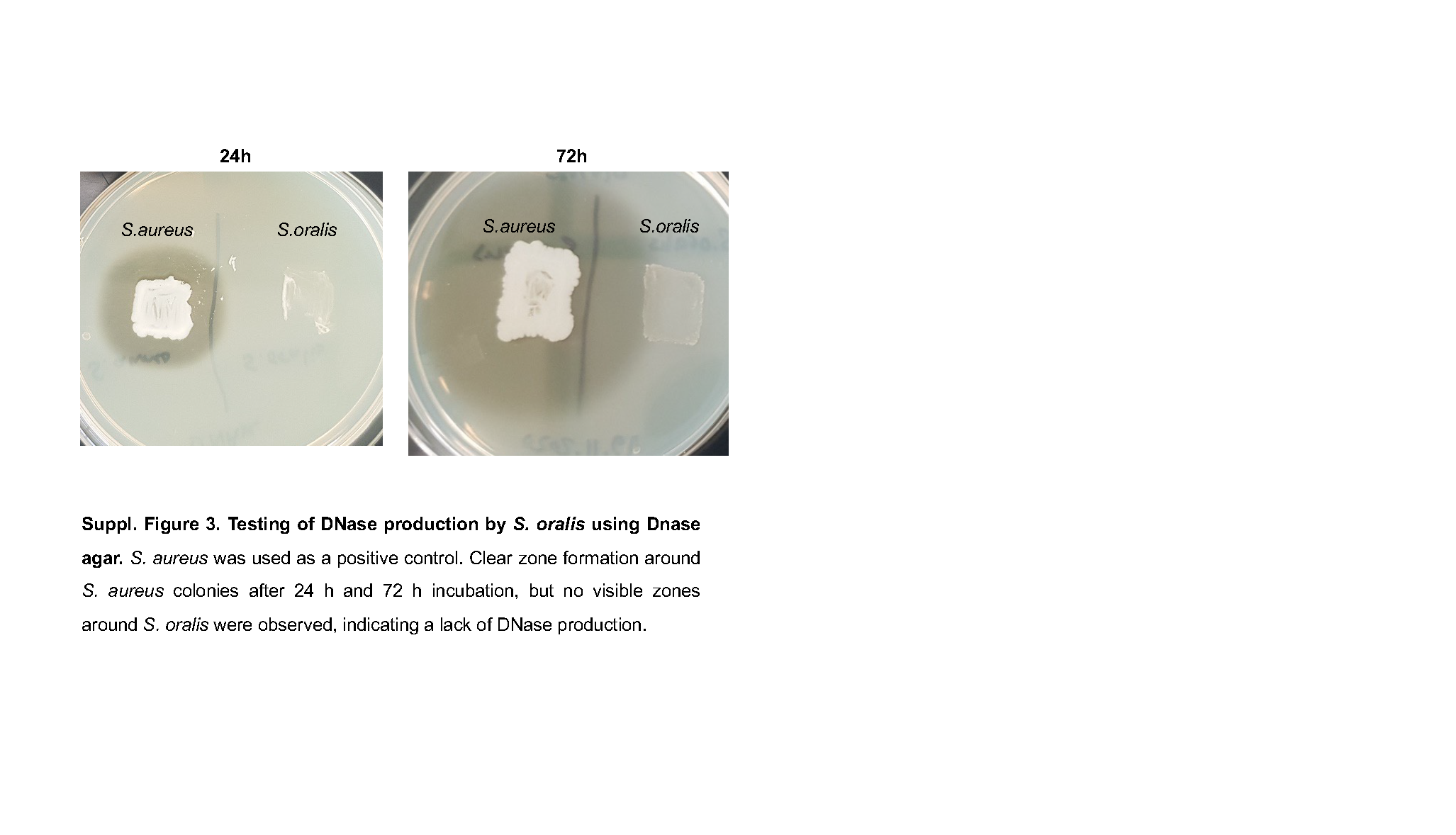


**Suppl. Figure 3. Testing of DNase production by *S. oralis* using Dnase agar.** *S. aureus* was used as a positive control. Clear zone formation around *S. aureus* colonies after 24 h and 72 h incubation, but no visible zones around *S. oralis* were observed, indicating a lack of DNase production.
